# Effects of Urate-Lowering Therapy on Risk of Hyperlipidemia in Gout by a Population-Based Cohort Study and on *In Vitro* Hepatic Lipogenesis-Related Gene Expression

**DOI:** 10.1155/2020/8890300

**Published:** 2020-11-14

**Authors:** Yi-Jen Fang, Tien-Yuan Wu, Cheng-Li Lin, Chih-Yang Su, Jia-Rong Li, Yun-Lung Chung, Ni Tien, Yun-Ping Lim

**Affiliations:** ^1^Research Center for Environmental Medicine, Kaohsiung Medical University, Kaohsiung, Taiwan; ^2^Ph.D. Program in Environmental and Occupational Medicine, College of Medicine, Kaohsiung Medical University and National Health Research Institutes, Taiwan; ^3^Graduate Institute of Clinical Medicine, Department of Environmental Health, Kaohsiung Medical University, Kaohsiung, Taiwan; ^4^National Institute of Environmental Health Sciences, National Health Research Institutes, Zhunan, Taiwan; ^5^Digestive Disease Center, Show Chwan Memorial Hospital, Changhua, Taiwan; ^6^Department of Pharmacy, Taichung Tzu Chi Hospital, Buddhist Tzu Chi Medical Foundation, Taichung, Taiwan; ^7^Department of Pharmacology, School of Medicine, Tzu Chi University, Hualien, Taiwan; ^8^Management Office for Health Data, China Medical University Hospital, Taichung, Taiwan; ^9^Master Program for Pharmaceutical Manufacture, College of Pharmacy, China Medical University, Taichung, Taiwan; ^10^Research Assistant Center, Show Chwan Health Care System, Changhua, Taiwan; ^11^Department of Medical Research and Development, Chang Bing Show Chwan Memorial Hospital, Changhua, Taiwan; ^12^Department of Laboratory Medicine, China Medical University Hospital, Taichung, Taiwan; ^13^Department of Medical Laboratory Science and Biotechnology, China Medical University, Taichung, Taiwan; ^14^Department of Pharmacy, College of Pharmacy, China Medical University, Taichung, Taiwan; ^15^Department of Internal Medicine, China Medical University Hospital, Taichung, Taiwan; ^16^Department of Medical Research, China Medical University Hospital, Taichung, Taiwan

## Abstract

Patients with gout are at a higher risk of cardiovascular disease, which is associated with hyperlipidemia. Management of gout in Taiwan is poor, and the association between urate-lowering therapy (ULT) among gout patients and hyperlipidemia is unclear. We conducted a retrospective cohort study using data from the Longitudinal Health Insurance Database (LHID) of Taiwan on new-onset gout patients and a comparison cohort without gout. A Cox proportional hazards model was used to analyze differences in the risk of hyperlipidemia between patients with and without gout after considering related comorbidities. We also examined the ULT medications on the hepatic expression of lipogenesis-related genes. After adjusting for potential confounders, the case group (44,413 patients) was found to have a higher risk of hyperlipidemia than the control cohort (177,652 patients) [adjusted hazards ratio (aHR) = 2.55]. Gout patients without antigout treatment had significantly higher risk of hyperlipidemia than the control cohort (aHR = 3.10). Among gout patients receiving ULT, except those receiving probenecid (aHR = 0.80), all had significantly lower risk of hyperlipidemia than gout patients without ULT (all aHR < 0.90). Using real-time polymerase chain reaction, we found that most of the antigout drugs decreased the expression of hepatic genes related to lipogenesis in differentiated HepaRG cells. These data indicate that these antigout drugs reduce hyperlipidemia in gout patients, partly via the reduction in expression of lipogenesis-related genes, leading to improved blood lipid profiles. We provide evidence of the strong association between gout and hyperlipidemia and highlight the need for appropriate treatment guidelines.

## 1. Introduction

Gout is a common form of severe, inflammatory arthritis in adults, especially in males [1]. It is characterized by the crystallization of uric acid (UA), a product of purine metabolism, as a result of chronic elevation of serum UA levels above the saturation point for urate crystal formation. Urate crystal deposits accumulate within small and large joints and may lead to irreversible dysfunction of peripheral joints, subcutaneous tophi, urate nephropathy, and urolithiasis [1, 2]. The disease burden is large and is likely to continue to increase in developed countries, affecting 0.53–6.1% of the population due to changes in lifestyle and diet [3].

Gout-related comorbidities and their treatment may have an effect on the development of gout and on the choice of therapeutic agents for gout. Drugs used to treat acute attacks of gout-related pain and prevent future pain attacks include nonsteroidal anti-inflammatory drugs (NSAIDs), consisting of over-the-counter options for an acute attack; colchicine for an acute attack or prescribed at a low daily dose to prevent future attacks; and corticosteroids to control gout inflammation and pain for patients who cannot take NSAIDs or colchicine [4]. However, urate-lowering therapy (ULT) may be recommended for patients who frequently experience gout attacks and to reduce gout-related complications. It includes xanthine oxidase inhibitors (XOIs) such as allopurinol and febuxostat, to block and lower UA production, and uricosurics such as benzbromarone, sulfinpyrazone, and probenecid, to remove UA from the kidneys [4].

A meta-analysis revealed that gout was associated with an increased risk of myocardial infarction (MI) [relative risk (RR) 1.45; 95% confidence interval (CI), 1.19–1.75] in both men and women [5]. In addition, a gradual increase in risk of MI was observed for younger age at gout onset (age 20–44 years, RR 2.82; 95% CI, 1.38–5.79) [5]. Gout was associated with an excess risk of cardiovascular disease- (CVD-) related mortality [hazard ratio (HR) 1.29, 95% CI 1.14–1.44] and coronary heart disease- (CHD-) related mortality (HR 1.42, 95% CI 1.22–1.63), which remained statistically significant after adjusting for traditional cardiovascular risk factors in a meta-analysis report [6]. Epidemiological studies originating from big data in healthcare support the association between gout and CVD. The prevalence of gout in the general population of Taiwan is 1 in 16 people; although the incidence has decreased recently, the prevalence remains unchanged [7]. Furthermore, asymptomatic hyperuricemia in elderly Chinese subjects without comorbidities was also linked to a significant risk of stroke events [8].

Several clinical reports have shown that hypercholesterolemia and hypertriglyceridemia are associated with an increased risk of gout [9, 10]. Hyperuricemia is an independent risk factor associated with the prevalence and parameters of metabolic syndrome [8, 11]. Hyperlipidemia [low levels of high-density lipoprotein cholesterol (HDL-C), high levels of triglycerides (TGs), total cholesterol (TC), and low-density lipoprotein cholesterol (LDL-C)] is one of the main characteristics of metabolic syndrome. This could be associated with higher incidence of stroke and CVD [12] as observed in an animal model [13].

A high LDL-C level is a strong risk factor for CVD, and hence, determining its risk factors is clinically relevant as it is an important therapeutic target for preventing CVD [14, 15]. Approximately 25–60% of patients with gout have higher levels of serum TC and TGs [16]; thus, gout control may be beneficial for blood lipid control. However, despite the high prevalence of gout, well-understood pathogenesis, and well-established ULT, gout is still ignored by doctors in primary care [2], who often treat gout as an acute disease, rather than as a chronic disease with major adverse consequences. Management of gout in Taiwan is poor, with only 1/5 affected patients being treated with ULT [7].

Liver X receptors (LXRs) are members of a nuclear receptor superfamily that function as ligand-activated transcription factors. There are two isoforms of LXRs, LXR*α* (encoded by *NR1H3*) and LXR*β* (encoded by *NR1H2*) [17]. LXR*β* is expressed ubiquitously, whereas LXR*α* is highly and specifically expressed in tissues with high levels of lipid metabolism that are responsible for *de novo* synthesis and excretion of cholesterol, such as the liver, adipose tissue, intestine, macrophages, and lungs [17]. Activation of LXR*α* results in the development of steatosis, which is mediated by the hepatic lipogenic pathway, primarily through sterol regulatory element binding protein 1 (SREBP-1c) [18]. In addition, hepatic expression of *LXRα*, *SREBP-1c*, and their target genes, such as acetyl-CoA carboxylase (*ACC*), fatty acid synthase (*FAS*), and stearoyl-CoA desaturase (*SCD*), has been found to be significantly upregulated in liver biopsies from nonalcoholic fatty liver disease (NAFLD) patients [19]. Ultrasonographic monitoring revealed hepatic steatosis in approximately 50% of hyperlipidemia patients, with hypertriglyceridemia being frequently associated with this condition [20]. Patients with NAFLD had significantly higher levels of LDL-C, TG, and TC, but lower levels of HDL-C; indeed, the increase in TG levels was positively related to the severity of NAFLD [20]. Thus, the most important role of LXR*α* is the maintenance of lipid homeostasis as it regulates the balance of lipid-metabolism genes.

As the relationship between gout and hyperlipidemia is not fully elucidated in Asian populations and the Nutrition and Health Survey in Taiwan reported prevalence of gout in 8.2% of men and 2.3% of women in Taiwan from 2005 to 2008 [21], it is important for physicians to understand the relationship between gout and hyperlipidemia. The objective of our study was to investigate the relationship between gout and hyperlipidemia by assessing the risk of development of hyperlipidemia in gout patients using the data obtained from the Taiwan National Health Insurance Research Database (NHIRD), which contains comprehensive information on diagnoses, prescriptions, and hospitalization of the entire population in Taiwan. Using this database, we conducted a population-based cohort study with a large sample drawn from Taiwanese adults and characterized the *in vitro* effects of ULT medications on the hepatic expression of lipogenesis-related genes that may play important roles in blood lipid homeostasis.

## 2. Materials and Methods

### 2.1. Data Source

The NHIRD was established in 1995 by the National Health Insurance Administration, Ministry of Health and Welfare, and consists of comprehensive information covering more than 99% insured persons who reside in Taiwan; it is also known as the Longitudinal Health Insurance Database (LHID). Between March 1995 and the end of 2013, the LHID included registration files and claim data on demographics, dates of clinical visits, and details of disease diagnoses and medical procedures for approximately 28.75 million (living and deceased) beneficiaries registered in the database. Upon request, the National Health Research Institute (the data holder of NHIRD) randomly selected a sample comprising one million individuals with a diagnosis of gout from NHIRD for use in this cohort study. This sample contained all original claim data of outpatient departments, emergency departments, and hospitalization.

Denominator data were based on the Registry of Beneficiaries, a part of NHIRD with records of the demographics, insurance status, residence, and socioeconomic data of all beneficiaries. However, ethnicity data were not available. The data were anonymized and deidentified prior to analysis; therefore, the need for patient consent was waived. Diagnostic codes were defined based on the International Classification of Diseases, 9^th^ Revision, Clinical Modification (ICD-9-CM) codes. The Research Ethics Committee of China Medical University Hospital in Taiwan approved the study (CMUH-104-REC2-115-R3).

### 2.2. Study Population

The date of diagnosis of gout was taken as the index date, and a total of 44,413 patients, who were diagnosed with gout (ICD-9-CM: 274) more than once in outpatient visits or at least once in inpatient visits and who were without hyperlipidemia (ICD-9-CM: 272) before the index date, between January 1, 2000, and December 31, 2013, were identified as the case cohort. Each case was randomly matched with 4 patients without gout during the same study period, and a total of 177,652 patients were recognized as the comparison cohort. Matching variables included sex, age, and index date, and we adopted two study cohorts by frequency matching on age with every 5-year span; hence, the index date of each sample in the comparison cohort was identical to the index date of the corresponding case. The end date of the follow-up period was the date on which hyperlipidemia occurred in patients; patients died or withdrew from NHIRD, or December 31, 2013, whichever was earlier.

The covariates of comorbidity comprised hypertension (ICD-9-CM: 401 to 405), stroke (ICD-9-CM: 430 to 438), diabetes mellitus (ICD-9-CM: 250), chronic obstructive pulmonary disease (COPD) (ICD-9-CM: 491, 492, and 496), coronary artery disease (CAD) (ICD-9-CM: 410 to 414), alcohol-related illness (ICD-9-CM: 291, 303, 305, 571.0, 571.1, 571.2, 571.3, 790.3, and V11.3), and asthma (ICD-9-CM: 493). Patients diagnosed with any of these comorbidities more than once in outpatient visits or at least once in inpatient visits before the index date were included in the study. Antigout treatments were determined by their Anatomical Therapeutic Chemical classification codes. The medications included in the analysis that available in Taiwan were allopurinol, febuxostat, benzbromarone, sulfinpyrazone, probenecid, and colchicine.

### 2.3. Chemical and Cell Culture

All chemicals were purchased from Sigma-Aldrich (St. Louis, Missouri, USA) and were of the highest-purity grade available. Chemicals were dissolved in dimethyl sulfoxide (DMSO) at appropriate concentrations before use. Human hepatoma HepaRG™ cells were purchased from Thermo Fisher Scientific (Waltham, Massachusetts, USA). Frozen cells were thawed and maintained in Williams' E medium (Sigma-Aldrich, St. Louis, Missouri, USA) supplemented with 10% FetalClone™ II serum (Hyclone™, GE Healthcare, Chicago, Illinois, USA), 1 × L-glutamine, 5 *μ*g/mL human insulin, and 50 *μ*M hydrocortisone hemisuccinate without antibiotics for 2 weeks. Next, the medium was replaced with the aforementioned medium plus 2% DMSO for two more weeks to induce differentiated hepatocyte-like properties. Cells were cultured in a humidified atmosphere of 5% CO_2_ at 37°C. Cell viability was assessed using *p*-nitrophenylphosphate (PNPP) in an acid phosphatase (ACP) assay, as previously reported [22].

### 2.4. RNA Isolation and Quantitative Real-Time PCR (qRT-PCR) Analysis

To evaluate the effects of ULT (allopurinol, febuxostat, benzbromarone, sulfinpyrazone, probenecid, and colchicine) on hepatic lipogenesis-related gene expression, mRNA levels were measured. Total RNA was extracted from differentiated HepaRG under various treatment conditions using a Direct-zol™ RNA MiniPrep kit (ZYMO Research, Irvine, CA, USA) by following the manufacturer's protocol. The quantity and purity of RNA were confirmed by calculating the ratio of the absorbance at 260 nm to absorbance at 280 nm. Total RNA (1 *μ*g) was subjected to synthesis of first-strand cDNA using a MultiScribe™ reverse transcriptase kit (Thermo Fisher Scientific, Waltham, MA, USA). Expression of *SREBP-1c*, *SCD*, *FAS*, fatty acid elongase (*FAE*), adenosine 5′-triphosphate (ATP) citrate lyase (*ACLY*), *ACC*, *LXRα*, and *β*-actin was analyzed by qRT-PCR using Luminaris Color HiGreen qPCR master mix (Thermo Fisher Scientific, Waltham, MA, USA) in a StepOnePlus™ Real-Time PCR System by using standard procedures. Each pair of specific primers used for real-time PCR analysis is listed in Table 1. The amount of target cDNA in each sample was calculated by determining a fractional PCR threshold cycle number (Ct value). The relative mRNA levels were normalized to those of *β*-actin, and the target cDNA expression was calculated using 2^−(Ct target gene–Ct *β-*actin)^. Data are presented as fold change compared to the control group.

### 2.5. Statistical Analysis

For intercohort comparisons, Student's *t*-tests and chi-square tests were used for continuous variables and categorical variables, respectively. Cumulative incidence rates and curves of hyperlipidemia were estimated and plotted by the Kaplan-Meier method, and log-rank tests were used to compare differences in time-to-event distributions between case and comparison cohorts. Cox proportional hazards models were developed to calculate HRs with 95% CIs of hyperlipidemia in patients, with or without gout. Adjusted HRs were calculated with the following covariates: age, sex, and comorbidities of hypertension, stroke, diabetes, COPD, CAD, alcohol-related illness, and asthma, to evaluate the risk of hyperlipidemia between case and comparison cohorts.

Subanalyses stratified by sex, age group, comorbidity, and antigout group were also performed to assess the association of gout with the subsequent risk of hyperlipidemia. Two-sided significance levels of all tests were set at 0.05. All data were analyzed using SAS 9.4 software (SAS Institute Inc., Cary, NC, USA), and cumulative incidence curves were plotted using R software (R version 3.5.3, GNU package).

For *in vitro* studies, data obtained from separate measurements were reported as the mean ± standarderror (SE). The *P* value for each experimental comparison was determined using analysis of variance, followed by the Least Significant Difference test for multiple comparisons. All *P* values were determined relative to the vehicle control group, as indicated in the figures. All statistical analyses were performed using SPSS for Windows, version 20.0 (IBM SPSS, Armonk, NY, USA). A *P* value less than 0.05 was considered statistically significant.

## 3. Results

### 3.1. Baseline Characteristics: Demographic and Association Findings

In this study, approximately 22.5% of patients were female, and the remaining were male. Among patients with or without gout, 57.6%, 24.1%, and 18.3% of patients were in the ≤49-, 50–64-, and ≥65-year-old age groups, respectively. The average age in the case cohort was 47.9 ± 16.9 years, and the average age in the comparison cohort was and 47.3 ± 17.1 years. The percentages of patients with hypertension, stroke, diabetes, COPD, CAD, alcohol-related illness, and asthma in the case cohort were significantly different from those in the comparison cohort (all *P* < 0.001). Approximately 14.3%, 0.21%, 43.4%, 2.87%, 0.51%, and 50.6% of patients received allopurinol, febuxostat, benzbromarone, sulfinpyrazone, probenecid, and colchicine, respectively (Table 2).

**Figure 1:**
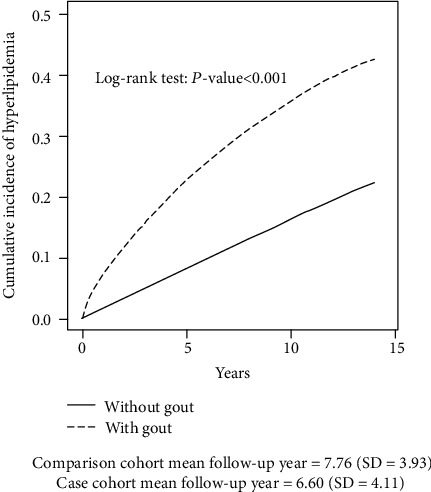
Comparison of cumulative incidence of hyperlipidemia between patients, with and without gout, using the Kaplan-Meier method. Comparison cohort mean follow-up year = 7.76 (SD = 3.93). Case cohort mean follow-up year = 6.60 (SD = 4.11).

The Kaplan-Meier curves are shown in Figure 1. The cumulative incidence of hyperlipidemia was lower in patients without gout than in patients with gout, and the log-rank test for the comparison of cumulative incidence curves revealed that *P* < 0.001. The average follow-up periods of the comparison and case cohorts were 7.76 ± 3.93 years and 6.60 ± 4.11 years, respectively.

Table 3 displays the incidence rates and HRs of hyperlipidemia in the case and comparison cohorts with different demographic characteristics. After adjustment, patients with gout were found to have significantly higher risk of developing hyperlipidemia than patients without gout (aHR = 2.55, 95% CI = 2.50–2.61, *P* < 0.001) regardless of sex (aHR = 2.15, 95% CI = 2.06–2.24, *P* < 0.001 in females; aHR = 2.70, 95% CI = 2.64–2.77, *P* < 0.001 in males), age (aHR = 3.08, 95% CI = 2.99–3.18, *P* < 0.001 in the ≤49 years' age group; aHR = 2.18, 95% CI = 2.10–2.26, *P* < 0.001 in the 50–64 years' age group; and aHR = 1.88, 95% CI = 1.79–1.98, *P* < 0.001 in the ≥65 years' age group), or comorbidity (aHR = 3.37, 95% CI = 3.28–3.46, *P* < 0.001 in the noncomorbidity group; aHR = 1.54, 95% CI = 1.49–1.60, *P* < 0.001 in the comorbidity group).

Table 4 shows the comparison among gout patients with and without antigout treatment and the comparison between nongout patients and gout patients without antigout treatment. Gout patients without antigout treatment showed significantly higher risk of hyperlipidemia than nongout patients (aHR = 3.10, 95% CI = 3.00–3.20, *P* < 0.001). Among gout patients, those with antigout treatment, except those receiving probenecid (aHR = 0.80, 95% CI = 0.64-1.00), had significantly lower risk of hyperlipidemia than gout patients without antigout treatment (aHR = 0.04, 95% CI = 0.01–0.17, *P* < 0.001 in patients receiving febuxostat; aHR = 0.57, 95% CI = 0.51–0.64, *P* < 0.001 in patients receiving sulfinpyrazone; aHR = 0.57, 95% CI = 0.54–0.61, *P* < 0.001 in patients receiving allopurinol; aHR = 0.89, 95% CI = 0.86–0.93, *P* < 0.001 in patients receiving benzbromarone; and aHR = 0.72, 95% CI = 0.68–0.76, *P* < 0.001 in patients receiving colchicine). In addition, among gout patients, those with different numbers of antigout combinations had significantly lower risk of hyperlipidemia than gout patients without antigout treatment (aHR = 0.86, 95% CI = 0.82–0.89, *P* < 0.001 in patients receiving one antigout treatment; aHR = 0.71, 95% CI = 0.68–0.75, *P* < 0.001 in patients receiving two combinations; aHR = 0.51, 95% CI = 0.47–0.55, *P* < 0.001 in patients receiving three combinations; and aHR = 0.35, 95% CI = 0.25–0.47, *P* < 0.001 in patients receiving four combinations of antigout treatments).

### 3.2. Effects of Antigout Drugs on Lipogenesis-Related Gene Expressions

**Figure 2:**
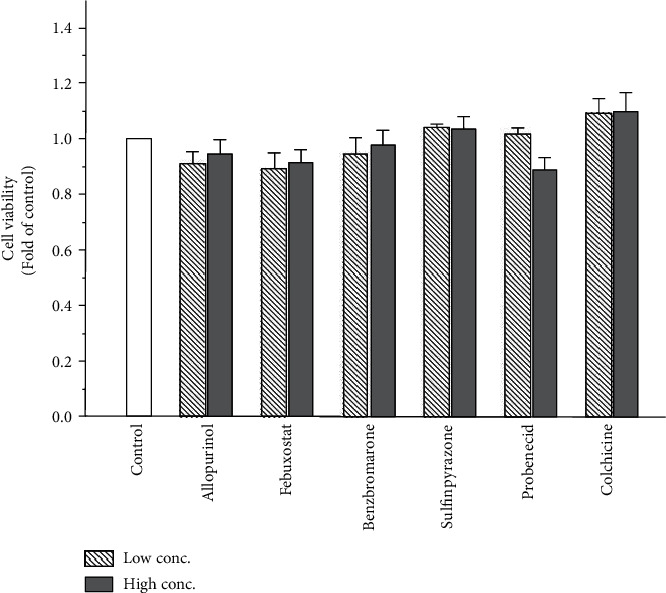
Viability of HepaRG cells following exposure to antigout drugs. HepaRG cells were exposed to allopurinol (14.7 and 22.1 *μ*M), febuxostat (5.66 and 13.09 *μ*M), benzbromarone (4.24 and 7.73 *μ*M), sulfinpyrazone (32.24 and 56.04 *μ*M), probenecid (243.9 and 520.6 *μ*M), and colchicine (7.51 × 10^−4^ and 0.075 *μ*M) for 24 h. Cell viability was monitored by measuring cellular acid phosphatase activity using *p*-nitrophenylphosphate as a substrate. Data shown are the mean ± standarderror (SE) (*n* = 3).

In this study, we also evaluated the effects of allopurinol, febuxostat, benzbromarone, sulfinpyrazone, probenecid, and colchicine on lipogenesis-related gene expression. The concentrations we used were based on the maximum serum drug concentrations [23–27]. As hepatic toxicity of these drugs is well-recognized [28], we examined their toxicity towards human hepatoma HepaRG cells in a cell viability assay. In this assay, the concentrations of these antigout drugs were 14.7 and 22.1 *μ*M (for allopurinol); 5.66 and 13.09 *μ*M (for febuxostat); 4.24 and 7.73 *μ*M (for benzbromarone); 32.24 and 56.04 *μ*M (for sulfinpyrazone); 243.9 and 520.6 *μ*M (for probenecid); and 7.51 × 10^−4^ and 0.075 *μ*M (for colchicine). HepaRG cells were treated with these antigout drugs for 24 h, followed by the ACP assay to assess their cytotoxicity. The ACP assay showed no significant difference in viability of control cells and cells treated with the antigout drugs (Figure 2).

**Figure 3:**
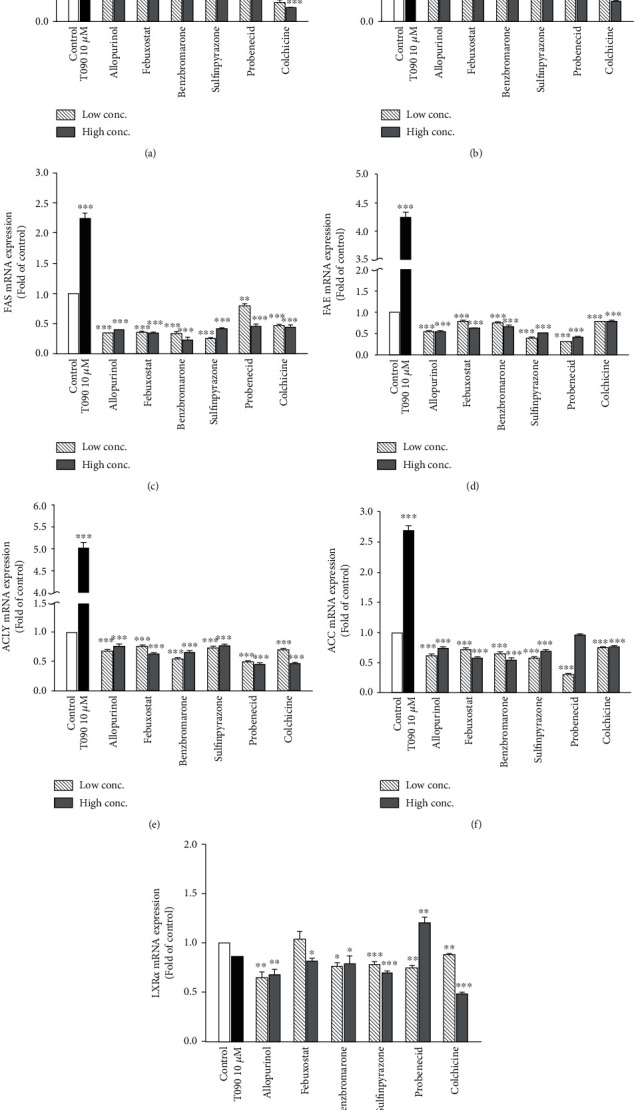
Expression of hepatic lipid metabolism-related genes following treatment with T0901317 and antigout drugs. Differentiated HepaRG cells were treated for 24 h with T0901317 (10 *μ*M), allopurinol (14.7 and 22.1 *μ*M), febuxostat (5.66 and 13.09 *μ*M), benzbromarone (4.24 and 7.73 *μ*M), sulfinpyrazone (32.24 and 56.04 *μ*M), probenecid (243.9 and 520.6 *μ*M), and colchicine (7.51 × 10^−4^ and 0.075 *μ*M). Following treatment, RNA was extracted, and the expression levels of (a) SREBP-1c, (b) SCD, (c) FAS, (d) FAE, (e) ACLY, (f) ACC, and (g) LXR*α* were analyzed by quantitative reverse transcription-polymerase chain reaction. Values were normalized to the expression of *β*-actin, with the *β*-actin levels of dimethyl sulfoxide- (DMSO-) treated cells set at 1. Results are expressed as the means ± standarderror (SE) (*n* = 3). ^∗^*P* < 0.05, ^∗∗^*P* < 0.01, and ^∗∗∗^*P* < 0.001 compared with cells treated with DMSO. SREBP-1c: sterol regulatory element binding protein 1; SCD: stearoyl-CoA desaturase-1; FAS: fatty acid synthase; FAE: fatty acid elongase; ACLY: adenosine 5′-triphosphate (ATP) citrate lyase; ACC: acetyl-CoA carboxylase; LXR*α*: liver X receptor alpha.

Expression of hepatic lipogenesis-related genes, *SREBP-1c*, *SCD*, *FAS*, *FAE*, *ACLY*, *ACC*, and *LXRα*, was measured in differentiated HepaRG cells by using real-time PCR. Differentiated HepaRG cells were treated with the antigout drugs for 24 h, total RNA was harvested, and expression of the indicated genes was analyzed. SREBP-1c is a hepatic transcription factor that regulates lipid homeostasis through several target lipogenic genes. T0901317 (T090), a synthetic LXR*α* agonist, significantly induces the expression of the target genes [29], *SREBP-1c*, *SCD*, *FAS*, *FAE*, *ACLY*, and *ACC*, and hence, was used as a positive control. However, mRNA levels for *SREBP-1c*, *SCD*, *FAS*, *FAE*, *ACLY*, *ACC*, and *LXRα* in the cells treated with most of these antigout drugs were significantly lower than those in the untreated cells, especially at the higher concentrations (Figures 3(a)–3(g)). However, *ACC* expression was not affected by the higher concentration of probenecid, while expression of *LXRα*, the master regulator of lipid homeostasis in hepatocytes, was induced by the higher concentration of probenecid. These data indicate that antigout treatment may partly reduce hepatic lipogenesis-related gene expression in gout patients, resulting in improved blood lipid profiles.

## 4. Discussion

To the best of our knowledge, there are few studies that have investigated the development of hyperlipidemia in gout patients and the medications used for gout therapy in Asian adults. Our study demonstrates the strong association between gout and the risk of hyperlipidemia by comprehensively adjusting for confounders. In this study, examining the correlation between gout and the risk of hyperlipidemia under antigout drug treatment, we observed the following: (1) we used a nationwide, population-based cohort study to comprehensively survey an Asian population and observed a strong association between gout and the incidence of hyperlipidemia; (2) overall incidence risk of hyperlipidemia in gout patients was 2.55-fold compared to that in nongout populations; (3) without ULT, the incidence of hyperlipidemia was higher in patients with gout than in patients without gout (aHR = 3.10, 95% CI = 3.00–3.20, *P* < 0.001); (4) with ULT, the risk for hyperlipidemia in gout patients was significantly lower when compared to gout patients without ULT (all aHR < 1); (5) using the hepatocyte-derived HepaRG cell line, we found that these antigout drugs significantly reduced the expression of lipogenic-related genes, *LXRα*, *SREBP-1c*, *SCD*, *FAS*, *FAE*, *ACLY*, and *ACC*, thus contributing to improved blood lipid profiles; (6) we investigated a single cohort consisting of a large number of participants for a long duration of the study period, and the outcome was adjusted for most confounding factors; (7) and physicians should be made aware of the benefits of antigout treatment for blood lipid levels and cardiovascular prognosis, so as to reduce the risk of CVD.

In a population-based study conducted in USA, researchers estimated that the annual incidence of gout was 0.45 (1977 to 1978), 0.62 (1995 to 1996), and 1.6 and 0.2 (men and women, respectively, 1948 to 1980) per 1000 person-years [30, 31]. In the UK, the overall gout incidence was estimated to be 1.4 per 1,000 person-years in 1981 [32], 1.31 in 1999 [33], and 1.77 in 2012 [34]. In contrast, the incidence of gout has rarely been estimated in populations of other ethnicities. Researchers from Taiwan had used the NHIRD to identify the prevalence and incidence of gout for each calendar year from 2005 to 2010 [7]. Of 23,371,362 beneficiaries in 2010, there were 1,458,569 prevalent and 56,595 incident cases of gout, giving a prevalence of 6.24% (95% CI = 6.23-6.25) and an incidence of 2.74 (95% CI = 2.72-2.76) per 1,000 person-years [7]. Among prevalent gout cases in 2010, only 22.93% (95% CI = 22.87-23.00) were prescribed ULT, suggesting that poor management of gout is a common problem in Taiwan.

Sustained reduction in serum UA levels is critical for the long-term management of gout. The recommended target serum UA level is <360 *μ*mol/L (or< 6.5 mg/dL), resulting in dissolution of monosodium urate crystals, suppression of acute gout pain, and resolution of gouty tophi [35]. Reduction in serum UA levels can be achieved by decreasing UA production with the use of XOIs, allopurinol and febuxostat; increasing excretion of UA with the use of uricosurics, benzbromarone, sulfinpyrazone, and probenecid; or metabolism of UA to the more water-soluble allantoin with the use of recombinant uricases, pegloticase and rasburicase (these two drugs were not available in Taiwan).

Uricosuric agents are more commonly prescribed than XOIs in Taiwan. In Taiwan, 1 in 16 people have gout, but only 1 in 5 affected people are treated with ULT [7]. Among patients who received ULT treatment, 60.08% received uricosuric agents alone, 28.54% received XOIs, and 11.38% received both. Our results found that 28.4% of gout patients did not receive any ULT, and 52.9%, 18.8%, and 28.3% received uricosuric agents, XOIs, and colchicine alone. As the database did not include nonprescribed drugs (such as NSAIDs), we focused on the aforementioned prescription drugs in our study.

Data from previous studies show that allopurinol, benzbromarone, and febuxostat can decrease blood levels of TC and TGs [36]. Thus, as statins and fenofibrate are expensive medications, these antigout drugs could facilitate the management of both gout and hyperlipidemia in gout patients. Therefore, we examined the effects of these drugs on the expression of hepatic lipogenesis-related genes.

A few studies have been conducted to investigate the effects of these antigout drugs on lipid metabolism regulation. New Zealand rabbits fed with a high fat diet showed significantly decreased serum TC and TG levels after administration of febuxostat for 4 weeks through antioxidant and anti-inflammatory mechanisms [37]. Lanaspa et al. [38] reported that high intracellular UA levels induce the mitochondrial translocation of the nicotinamide adenine dinucleotide phosphate (NADPH) oxidase subunit, NADPH oxidase 4 (Nox4), thus leading to increased oxidative stress and mitochondrial dysfunction and subsequent citrate release into the cytosol for *de novo* lipogenesis and TG synthesis [38]. In their study, they proposed that fructose induces UA production and TG synthesis; UA either alone or as a by-product generated by the initial phosphorylation of fructose, from 0 to 750 *μ*mol/liter (4–12 mg/dL), can also induce lipid synthesis independent of fructose. They challenged hepatocytes with fructose in the presence of allopurinol to block UA production; allopurinol effectively blocked the production of UA upon exposure of the cells to fructose. Although allopurinol may have off-target effects (due to its antioxidant properties), the prevention of fructose-induced lipogenesis appears to be dependent on its ability to reduce UA levels because the subsequent addition of UA restored the metabolic effects of fructose. Notably, TG accumulation was also inhibited by allopurinol, indicating that UA may play a key role in the lipogenic ability of fructose through the stimulation of NADPH oxidase. Consistent with this finding, UA also stimulates oxidative stress in the mitochondria and further decreases the mitochondrial membrane potential. Fructose-induced oxidative stress was blocked by allopurinol. High levels of UA (750*μ*mol/liter ≈ 12mg/dL) also induced significant changes in mitochondrial morphology as determined via electron microscopy. Compared with the controls, the UA-treated HepG2 cells exhibited mitochondria that were shorter and smaller, suggesting mitochondrial fragmentation [38]. The authors also found that lowering the intracellular UA levels could prevent fat accumulation in fructose-exposed HepG2 cells and thus might also prevent fatty liver in an animal model of hyperuricemia and metabolic syndrome. Allopurinol effectively reduced the intrahepatic UA levels, TG content, and hepatic cholesterol content. These findings corresponded with the significantly decreased activity of NOX4 in the mitochondria of mice receiving allopurinol [38]. The authors also determined whether hyperuricemia could predict NAFLD independent of obesity by examining a population undergoing hemodialysis, as hyperuricemia is common even when obesity is absent; they found that serum UA levels were associated with the increasing prevalence of hepatic steatosis independent of obesity [38]. Clinically, the UA in patients decreases upon challenge with antigout treatment; moreover, we found that antigout drugs reduce lipogenesis-related gene expression as well as the upper control elements, SREBP-1c and LXR*α*, suggesting that these drugs can affect earlier gene expression. These dual effects of antigout treatments, direct decreases in lipogenesis gene expression and UA production and, thus reduced lipogenesis might be beneficial for gout patients.

Furthermore, XO-mediated conversion of hypoxanthine/xanthine to UA is associated with the generation of hydrogen peroxide [39], and UA forms reactive intermediates, including alkylating species, by reacting with peroxynitrite [40]. Other potential mechanisms have been proposed to explain how UA can modulate LDL-C levels, which are ameliorated by the XOI, allopurinol, by decreasing lipid peroxidation rates [41]. Nevertheless, the exact mechanism by which UA increases LDL-C and TG levels is still unclear.

The most frequently used urate-lowering drug, allopurinol, is effective, easy to administer (once-daily dosing), inexpensive, and generally well-tolerated. However, it usually causes severe cutaneous adverse reactions, such as hypersensitivity syndrome and Stevens-Johnson syndrome, and is associated with human leukocyte antigen HLA-B∗5801, especially in Han Chinese (20% of Han Chinese population are HLA-B∗5801 carriers) [42]. As suggested by the Taiwan Department of Health, we applied the molecular diagnosis of the patients' genotype before allopurinol administration so as to reduce the side effects of this drug. Such recommendations have been made about the patients' genotype before drug administration. Moreover, allopurinol has been associated with a significant reduction in CVD-related mortality (RR = 0.74, 95% CI = 0.61–0.90) [43] and is also beneficial in ischemic heart disease patients [44].

Another XOI, febuxostat, was approved by the European Medicines Agency in 2008 and the United States Food and Drug Administration in 2009 for the treatment of gout [35]. It has shown a superior urate-lowering effect compared to allopurinol, but information on its effects on blood cholesterol or TGs in gout patients is limited [45].

Benzbromarone, sulfinpyrazone, and probenecid lower serum UA levels by increasing renal urate excretion. Benzbromarone is not available in many countries because of concerns about its hepatotoxicity, and it is not recommended for patients with liver and renal dysfunction [46]. This risk could be reduced by increasing the dose gradually and monitoring liver function. Our results reveal that viability of HepaRG cells was not significantly affected by the concentration of benzbromarone that is equivalent to its Cmax concentration in humans. However, benzbromarone reduced the expression of *LXRα* and its target lipogenic-related genes. In contrast, probenecid induced the expression of *LXRα* and did not affect gene expression of *ACC* even at a higher concentration. Thus, benzbromarone may contribute to the lower risk of hyperlipidemia in gout patients in our study.

Colchicine is another commonly used drug for acute gout treatment. In the Acute Gout Flare Receiving Colchicine Evaluation study, although low- (1.8 mg total) and high- (4.8 mg total) dose colchicine regimens were effective in pain reduction, low-dose colchicine had fewer adverse effects [47]. Gout patients often have related medical conditions that may promote the development of gout and make treatment more challenging. The treatment options for acute gout and prevention of gout need to be adjusted based on patients' comorbidities and their coprescribed drugs.

Hyperuricemia is frequently reported in patients with CVD and is associated with CVD-related mortality, suggesting that it may have a direct vascular effect [48, 49]. A previous report on hypercholesterolemia and hypertriglyceridemia patients treated with febuxostat, allopurinol, and benzbromarone revealed that febuxostat significantly decreased serum TC and TG levels, whereas allopurinol and benzbromarone only modestly decreased TG levels, but not TC levels, especially after 3–5 weeks of treatment [36]. No serious adverse events were observed. Our study included a long follow-up period of up to 6.60 and 7.76 years for the case and control groups, respectively, which may have increased the likelihood of observing hyperlipidemia treatment with antigout drugs significantly reduced the risk of hyperlipidemia.

This study has some strengths that are noteworthy. As this study was based on a large and representative population cohort extracted from the Taiwan NHI system, it eliminates bias from selection, nonresponse, or poor recall. The LHID has been shown to have good levels of accuracy and completeness in recording prescriptions and clinical diagnoses. In addition, we adjusted for many potentially confounding factors that are associated with hyperlipidemia and selected 4 times the number of nongout patients to form the control group. Both the case and control groups were studied for a long observation period. This study shows that gout patients had a higher risk of hyperlipidemia with a narrower and statistically significant CI.

This is the first study to examine an Asian population by using a population-based, nationwide cohort study to evaluate the effects of antigout therapy on hyperlipidemia in gout patients. However, our study has several limitations. First, patients' information concerning lifestyle, smoking status, alcohol consumption, environmental exposure, body mass index, and family history of hyperlipidemia was unavailable in the LHIRD. The aforementioned factors may contribute as confounding factors to the development of hyperlipidemia. LHIRD claims data are used mainly for administrative billing purposes. Therefore, the additional information was anonymous, and it was not possible to directly contact the patients for additional information. Second, no data on diets were available in this study, and dietary intake may contribute to the patients' lipid profiles. Third, exact laboratory data in the LHIRD were limited; therefore, we did not know the serum UA status in gout patients after ULT treatment. Fourth, the LHIRD lacks complete information regarding patients' liver disease by Child-Pugh classification, which might highly correlate with the development of hyperlipidemia. Therefore, our study reduced the confounding effects of the medications by adjusting for comorbidities. In addition, the severity of gout and disease duration may also affect blood lipid levels. This is an epidemiological study that can only provide evidence to show the association between gout and hyperlipidemia and cannot show a causal relationship. It is necessary to obtain more information from other databases or questionnaires to conduct a prospective study or randomized controlled trial to investigate such a relationship between gout, ULT, and hyperlipidemia. Therefore, the essential part of the optimization of care of patients with gout is likely to be physician education to improve knowledge and promote interest in gout. However, our results provide evidence that recommendations and guidelines for clinical management of gout patients can effectively mitigate long-term consequences of hyperlipidemia such as the possibility of developing CVD. Although we cannot identify the levels of each type of lipid or the levels of serum UA, we identified gout patients without ULT who may have had high levels of UA, which could be correlated with high serum LDL-C, TG, and TC levels and with low levels of HDL-C. Peng et al. identified HDL-C as a protective factor for CVD risk, which is inversely correlated to serum UA levels, but positively correlated with levels of LDL-C and TGs [8]. Thus, their findings suggest that a greater atherogenic ability is linked to a predisposition to CVD. Our study indicates that gout might intensify many pathophysiological mechanisms associated with the development of hyperlipidemia and consequently CVD. Thus, treatment of gout with ULT might be also beneficial for blood lipid levels through the inhibition of lipogenesis-related gene expression.

## 5. Conclusion

This population-based cohort study demonstrates a substantial association between gout, ULT, and the risk of hyperlipidemia. We confirm that management of gout in Taiwan remains poor, with 28.4% of gout patients not being prescribed ULT, which could contribute to elevated serum UA levels and increased gout flares with major adverse consequences such as hyperlipidemia. Due to the strong association between gout and hyperlipidemia, appropriate treatment guidelines such as changes in lifestyle, diet, and measurement of serum parameters should be developed to improve serum UA levels and understand the therapeutic mechanism of action of these drugs. We provide evidence of the benefits of these common antigout drugs for hyperlipidemia in gout patients through the reduction in the expression of lipogenesis-related genes.

## Figures and Tables

**Table 1:** Sequences of PCR primers.

Gene	Species	Forward primer (5′-3′)	Reverse primer (5′-3′)
*SREBP-1c*	Human	CGC TCC TCC ATC AAT GAC AA	TGC AGA AAG CGA ATG TAG TCG AT
*SCD*	Human	CCG ACG TGG CTT TTT CTT CT	GCG TAC TCC CCT TCT CTT TGA C
*FAS*	Human	ACA TCA TCG CTG GTG GTC TG	GGA GCG AGA AGT CAA CAC GA
*FAE*	Human	TTC CGA GTC TCC CGG AAG T	ACA GCC CAT CAG CAT CTG AGT
*ACLY*	Human	GTG TGG ACG TGG GTG ATG TG	TTG ATG TCC TCA GGA TTC AGT TTC
*ACC*	Human	CTC TTG ACC CTG GCT GTG TAC TAG	TGA GTG CCG TGC TCT GGA T
*LXRα*	Human	CGA TC GAG GTG ATG CTT CTG	GGC AAA GTC TTC CCG GTT AT
*β-Actin*	Human	CCT GGC ACC CAG CAC AAT	GCC GAT CCA CAC GGA GTA CT

**Table 2:** Demographic characteristics, comorbidities, and medication in patients with and without gout.

Variable	Gout		*P* value
	No	Yes	
	*N* = 177652	*N* = 44413	
Sex	*N* (%)	*N* (%)	0.99
Female	39952 (22.5)	9988 (22.5)	
Male	137700 (77.5)	34425 (77.5)	
Age, mean (SD)^#^	47.3 (17.1)	47.9 (16.9)	<0.001
Stratified age			0.99
≤49	102312 (57.6)	25578 (57.6)	
50-64	42884 (24.1)	10721 (24.1)	
65+	32456 (18.3)	8114 (18.3)	
Comorbidity			
Hypertension	19455 (11.0)	6396 (14.4)	<0.001
Stroke	6654 (3.75)	2063 (4.65)	<0.001
Diabetes	4786 (2.69)	1674 (3.77)	<0.001
COPD	8137 (4.58)	2507 (5.64)	<0.001
CAD	7870 (4.43)	2839 (6.39)	<0.001
Alcohol-related illness	7922 (4.46)	2728 (6.14)	<0.001
Asthma	5049 (2.84)	1723 (3.88)	<0.001
Medication			
Allopurinol		6346 (14.3)	
Febuxostat		93 (0.21)	
Benzbromarone		19286 (43.4)	
Sulfinpyrazone		1275 (2.87)	
Probenecid		226 (0.51)	
Colchicine		22482 (50.6)	

Chi-square test. ^#^Student's *t*-test.

**Table 3:** Comparison of incidence and hazard ratio of hyperlipidemia stratified by sex, age, and comorbidity between patients with and without gout.

Variable	Gout						Crude HR (95% CI)	Adjusted HR^†^ (95% CI)
	No			Yes				
	Event	PY	Rate^#^	Event	PY	Rate^#^		
All	24485	1378799	17.8	13639	293172	46.5	2.61 (2.55, 2.66)^∗∗∗^	2.55 (2.50, 2.61)^∗∗∗^
Sex								
Female	7080	282065	25.1	3217	59166	54.4	2.14 (2.06, 2.23)^∗∗∗^	2.15 (2.06, 2.24)^∗∗∗^
Male	17405	1096734	15.9	10422	234006	44.5	2.80 (2.73, 2.87)^∗∗∗^	2.70 (2.64, 2.77)^∗∗∗^
Stratified age								
≤49	10087	850974	11.9	7382	191709	40.6	3.44 (3.34, 3.55)^∗∗∗^	3.08 (2.99, 3.18)^∗∗∗^
50-64	9290	312176	29.8	4160	63233	65.8	2.19 (2.11, 2.27)^∗∗∗^	2.18 (2.10, 2.26)^∗∗∗^
65+	5108	215649	23.7	2097	48229	43.5	1.82 (1.73, 1.91)^∗∗∗^	1.88 (1.79, 1.98)^∗∗∗^
Comorbidity^‡^								
No	13645	1003452	13.6	10840	375347	28.9	3.40 (3.31, 3.49)^∗∗∗^	3.37 (3.28, 3.46)^∗∗∗^
Yes	8639	181008	47.7	5000	112164	44.6	1.57 (1.52, 1.62)^∗∗∗^	1.54 (1.49, 1.60)^∗∗∗^

PY: person-years; Rate^#^: incidence rate, per 1,000 person-years; Crude HR: crude hazard ratio; Adjusted HR^†^: multivariable analysis including age, sex, and comorbidities of hypertension, stroke, diabetes, COPD, CAD, alcohol-related illness, and asthma; Comorbidity^‡^: patients with any one of the comorbidities of hypertension, stroke, diabetes, COPD, CAD, alcohol-related illness, and asthma were classified as the comorbidity group. ^∗∗∗^*P* < 0.001.

**Table 4:** Incidence, crude, and adjusted hazard ratio of hyperlipidemia compared among gout patients with or without antigout treatment and compared between gout patients without antigout treatment and nongout patients.

Variables	*N*	Event	PY	Rate^#^	Crude HR (95% CI)	Adjusted HR^†^ (95% CI)	Adjusted HR^†^ (95% CI)
Nongout	177652	24485	1378799	17.8	1	1	
Gout without the selected antigout treatment	12617	4279	73332	58.4	3.26 (3.15, 3.36)^∗∗∗^	3.10 (3.00, 3.20)^∗∗∗^	1
Gout with antigout treatment							
Febuxostat	93	2	860	2.33	0.13 (0.03, 0.53)^∗∗^	0.12 (0.03, 0.47)^∗∗^	0.04 (0.01, 0.17)^∗∗∗^
Probenecid	226	77	1810	42.6	2.40 (1.92, 3.00)^∗∗∗^	2.42 (1.93, 3.02)^∗∗∗^	0.80 (0.64, 1.00)
Sulfinpyrazone	1244	301	9579	31.4	1.77 (1.58, 1.98)^∗∗∗^	1.74 (1.55, 1.95)^∗∗∗^	0.57 (0.51, 0.64)^∗∗∗^
Allopurinol	5885	1459	46659	31.3	1.76 (1.67, 1.86)^∗∗∗^	1.70 (1.62, 1.80)^∗∗∗^	0.57 (0.54, 0.61)^∗∗∗^
Benzbromarone	15352	5193	103671	50.1	2.81 (2.73, 2.89)^∗∗∗^	2.78 (2.69, 2.86)^∗∗∗^	0.89 (0.86, 0.93)^∗∗∗^
Colchicine	8996	2328	57261	40.7	2.27 (2.18, 2.37)^∗∗∗^	2.29 (2.19, 2.39)^∗∗∗^	0.72 (0.68, 0.76)^∗∗∗^
Number of antigout treatments							
1	17125	5314	107897	49.3	2.76 (2.67, 2.84)^∗∗∗^	2.67 (2.59, 2.75)^∗∗∗^	0.86 (0.82, 0.89)^∗∗∗^
2	11670	3359	85575	39.3	2.21 (2.13, 2.29)^∗∗∗^	2.22 (2.14, 2.30)^∗∗∗^	0.71 (0.68, 0.75)^∗∗∗^
3	2764	646	24083	26.8	1.52 (1.40, 1.64)^∗∗∗^	1.53 (1.41, 1.65)^∗∗∗^	0.51 (0.47, 0.55)^∗∗∗^
4	234	41	2252	18.2	1.03 (0.76, 1.40)	1.02 (0.75, 1.38)	0.35 (0.25, 0.47)^∗∗∗^
5	3	0	34	0.00	—	—	—

PY: person-years; Rate^#^: incidence rate, per 1,000 person-years; Crude HR: crude hazard ratio; Adjusted HR^†^: multivariable analysis including age, sex, and comorbidities of hypertension, stroke, diabetes, COPD, CAD, alcohol-related illness and asthma. ^∗∗^*P* < 0.01; ^∗∗∗^*P* < 0.001.

## Data Availability

The data used to support the findings of this study are included within the article.
